# Ruxolitinib in patients with graft versus host disease (GvHD): findings from a compassionate use program

**DOI:** 10.1038/s41409-024-02207-4

**Published:** 2024-02-15

**Authors:** Thirupathi Pattipaka, Séverine Sarp, Peyman Nakhaei, Sibel Güneş

**Affiliations:** 1 Novartis Pharma AG, Basel, Switzerland; 2 Novartis Pharmaceuticals Canada Inc, Montreal, QC Canada

**Keywords:** Graft-versus-host disease, Molecularly targeted therapy

## Abstract

The ruxolitinib compassionate use (CU) program offered ruxolitinib to patients ≥2 years of age with confirmed steroid-resistant acute or chronic graft-versus-host disease (aGvHD and cGvHD, respectively). Data from 1180 patients (*n* = 775, 370 and 35 with cGvHD, aGvHD, and non-specified GvHD, respectively) were analyzed. Most patients had severe cGvHD (56%) or stage III/IV aGvHD (70%) disease and had previously received corticosteroids ( > 80%); ruxolitinib was requested primarily as a second-/third-line option. Patients <12 and ≥12 years old most often received the recommended ruxolitinib doses (5 mg twice daily [BID] and 10 mg BID, respectively); however, 23% and 30% of ≥12 year olds with cGvHD and aGvHD, respectively, received the lower dose of 5 mg BID. Notably, corticosteroid usage decreased with ruxolitinib treatment; at the initial ruxolitinib request, 81% and 91% of patients with cGvHD and aGvHD, respectively, were receiving corticosteroids whereas at resupply, 62% and 64%, respectively, were receiving corticosteroids. Eighty two percent of evaluable patients with cGvHD had a complete or partial response to treatment and 56% of evaluable patients with aGvHD had a best response of grade 0/I. These findings demonstrate the rapid and positive effects of ruxolitinib in patients with GvHD in a real-world setting.

## Introduction

Graft versus host disease (GvHD) is a life-threatening, multisystemic disorder and the main complication in patients who have undergone allogeneic hematopoietic stem cell transplantation (alloHSCT) [1], affecting general morbidity/quality of life (QoL) in its acute (aGvHD) and chronic (cGvHD) forms [2–5].

Systemic corticosteroids are the first-line treatment for aGvHD (Grade II or higher) and cGvHD (moderate to severe) [6–8]; however, there is an approximate 30–60% response rate to corticosteroid treatment in patients with aGvHD and half of patients with cGvHD require second-line treatment [9–11]. Unfortunately, no standard second-line therapy exists [8] and professional societies recommend that physicians follow their own institutional guidelines and include patients in clinical trials when possible [6–8].

Ruxolitinib is an oral, selective Janus kinase 1 and 2 (JAK1/JAK2) tyrosine kinase inhibitor approved for the treatment of patients aged 12 years and older with aGvHD or cGvHD who have an inadequate response to corticosteroids or other systemic therapies [12, 13]. These approvals were based on outcomes from the Phase III REACH2 and REACH3 trials, which demonstrated significantly higher overall response rates (ORR) with ruxolitinib versus best available treatment (BAT; REACH2 ORR at day 28: 62% vs 39%, respectively [*P* < 0.001]; REACH3 ORR at week 24: 49.7% vs 25.6% [*P* < 0.001], respectively) in patients with steroid-refractory aGvHD and steroid-refractory or -dependent cGvHD, respectively [14, 15].

For many patients with serious or life-threatening medical conditions like GvHD, waiting for drugs to be licensed in their country means unnecessary delays. When available treatment options have been exhausted and clinical trial enrollment is not an option, compassionate use (CU) programs can provide early access to locally unlicensed treatments [16, 17]. When early, positive findings were reported for ruxolitinib in patients with GvHD from the REACH studies [14, 15, 18], a CU program was established to provide global access to ruxolitinib for patients with confirmed steroid-refractory aGvHD and cGvHD. In the current study, patient data from this CU program were analyzed to describe clinical characteristics, dosing patterns, concomitant medications, and response to treatment to provide insights on the use of ruxolitinib for GvHD in a real-world setting.

## Methods

### Study design and patients

This was an observational, cohort study of data from a CU program for ruxolitinib in GvHD. Physicians applied for access to ruxolitinib through the Novartis online system (Managed Access Programs | Novartis) [17]. Initial applications were reviewed by Novartis medical personnel against pre-defined criteria; patients needed to be ≥2 years of age with a confirmed diagnosis of steroid-refractory aGvHD or cGvHD and evidence of myeloid and platelet engraftment (Supplementary Table 1 for full CU program eligibility criteria).

Eligible patients received an initial 3-month supply of ruxolitinib, after which physicians could apply for additional 3-month resupplies (every 90 days). Patients from Korea received a one-time 6-month supply of ruxolitinib only. The CU treatment plan recommended ruxolitinib doses of 10 mg twice daily (BID) for patients ≥12 years of age, 5 mg BID for patients ≥6 to <12 years of age and 4 mg/m^2^ for those ≥2 to <6 years of age. These recommended doses are consistent with those used in the REACH2 [14] and REACH3 [15] studies in patients ≥12 years of age, and REACH4 [19, 20] and REACH5 [21–23] studies conducted in pediatric patients. The dose administered was at the discretion of the treating physician and could be modified to allow continued participation, or tapered if a clinical response was demonstrated (Ruxolitinib dose modifications in supplementary materials). Concomitant medications, such as cytochrome 450 inhibitors/modulators, required modification of the ruxolitinib dose [12, 13]. Patients were included in this analysis if they had been treated with ruxolitinib at least once and were followed from index date (date of the first treatment of ruxolitinib for aGvHD or cGvHD) until their last resupply.

### Data collection

Physicians completed an assessment form at the initial CU request (baseline) that captured patient characteristics and disease management, and a separate form at each resupply request with complementary questions to the initial form plus questions regarding efficacy (Table 1**;** Supplementary Table 2 for full questions/options). Physicians’ resupply responses were checked daily to identify potential adverse events (AEs), which were followed-up with the physician; these AEs, and any other physician-reported AEs, were captured in the safety database, ARGUS, and analyzed separately (see Supplementary Table 3).

**Table 1:** Overview of the baseline and follow-up questions at resupply request for patients with aGvHD and cGvHD enrolled in the ruxolitinib CU program.

Steroid-refractory aGvHD	
Baseline questions	Resupply questions
Is your request for aGvHD?^a^	Is your request for aGvHD?^a^
Please tick the relevant boxes for organ involvement of aGvHD at present (skin, upper GI, lower GI, liver).^b^	Please tick the boxes for organ involvement at present (skin, upper GI, lower GI, liver).^b^
What is the overall grade of aGvHD at present (grade 0 to IV)?^b^ [20]	What is the overall grade of aGvHD at present?^b^ [20]
Which line of treatment is ruxolitinib going to be used for (1^st^ to beyond 4^th^)?^b^	Have you been modifying the dose of the corticosteroids (CS) since initiation of ruxolitinib?^b^
Please provide systemic therapy(ies) that are currently in use to treat aGvHD.^c^	Please provide systemic therapy(ies) used at present to treat aGvHD (in addition to ruxolitinib).^c^

Steroid-refractory cGvHD	
Baseline questions	Resupply questions
Is your request for cGvHD?^a^	Is your request for cGvHD?^a^
Has the patient presented with aGvHD prior?^a^	Please specify the overall response to ruxolitinib according to NIH Consensus for measuring therapeutic response.^b^ [19]
Which line of cGvHD treatment is ruxolitinib going to be used for (1^st^ to beyond 4^th^)?^b^	What is the overall severity of cGvHD at present (NIH)?^b^ [21]
Please provide systemic therapy(ies) that are currently in use to treat cGvHD^c^	Have you been modifying the dose of the corticosteroids (CS) since initiation of ruxolitinib?^b^
Please specify the overall response to the most recent line of treatment patient has received according to NIH Consensus for measuring therapeutic response.^b^ [19]	Please provide systemic therapy(ies) used at present to treat cGvHD (in addition to ruxolitinib).^c^
What is the overall severity of cGvHD at present (NIH)?^b^ [21]	

Note that this table provides only the overall questions that were asked. Footnotes identify the additional content and drop-down menus available: ^a^Yes/No options; ^b^Drop-down menu available; ^c^Multiselect menu available. See Supplementary Table 2 for a full list of options for the drop-down and multiselect menus. Follow-up questions at resupply were implemented in December 2019.

*aGvHD* acute graft versus host disease; *cGvHD* chronic graft versus host disease; *CU* compassionate use; *GI* gastrointestinal; *NIH* National Institutes of Health.

Data were collected at baseline (inclusion in the CU program), month 3 (after initial dose) and every 3 months thereafter with a ± 1-month window to accommodate follow-up reports from physicians. Patient demographics, country, disease grading and organ involvement, line of therapy, ruxolitinib dose, treatment duration, response during follow-up and prior treatments were extracted (Supplementary Table 4). Response, disease grading and organ involvement were assigned as per the options in Supplementary Table 2 [24–26].

### Study objectives

The primary objectives were to describe the demographics, clinical characteristics, and prior treatments of patients with steroid-refractory aGvHD and cGvHD who received ruxolitinib within the CU program. Secondary objectives included describing treatment patterns, ruxolitinib dose modifications, corticosteroid dose reductions or discontinuations, concomitant medications used, and disease progression through changes in disease grade and response. Reported AEs were analyzed and described.

### Ethics

Local regulatory or independent ethics committee approval was obtained for each patient treated in the ruxolitinib CU program, as per local regulations, in line with the ethical principles in the Declaration of Helsinki. Physicians obtained written informed consent from all patients or their legal guardians prior to the start of treatment.

### Statistical analysis

The current analysis was based on GvHD ruxolitinib requests approved between 1 January 2018 to 26 November 2021, which reflected the substantial number of CU requests received during this period rather than pre-determined, formal criteria. Distinct supplies and corresponding resupplies were assumed to be from a single patient. In cases of premature discontinuation due to withdrawal of consent, only the information collected prior to the discontinuation was analyzed; no imputation methods were used for missing data and cancelled requests were not included in the analysis. Data for aGvHD and cGvHD were analyzed separately. As details of the requests for a resupply or not were captured in the CU system, patients did not have defined stop dates for treatment and, therefore, the length of follow-up and treatment duration for patients could not be determined definitively. Patients who did not request resupply were considered ‘lost to follow-up’ and treatment duration was calculated based on an assumption that patients stopped treatment 45 days after their last resupply.

Data were captured as absolute values (e.g., age), distinct predetermined categories (e.g., stage of disease) (see Supplementary Table 2), and as open questions (e.g., dose). Categorical variables are summarized as number (n) of patients or percentage (%) of the total population and continuous variables as mean, median, standard deviation (SD) and interquartile ranges (IQR). Data were analyzed using R statistical programming tools (version 4.0.3; https://www.r-project.org/) and presented descriptively.

## Results

### Patients and CU requests

**Fig. 1:**
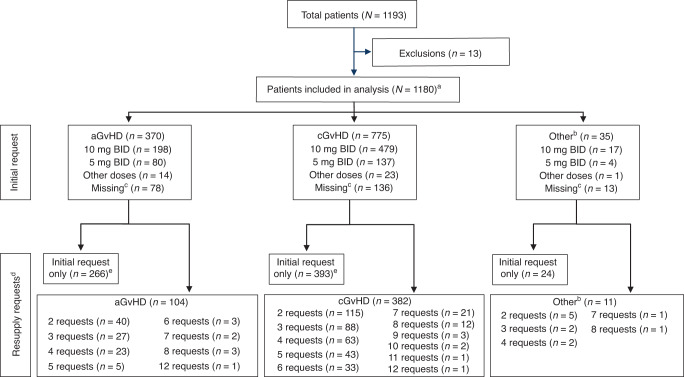
Patients initiating a CU request and resupplies. ^a^One patient classified as NA; ^b^Other types or non-specified GvHD; ^c^No information on the dose for these patients; ^d^Resupply requests denotes the number of patients who had 2–12 requests, including the initial request; ^e^Patients discontinued after the initial supply of ruxolitinib. Note that physicians did not provide reasons for not requesting resupplies, therefore, no data is available on why patients discontinued the program. aGvHD acute graft versus host disease, BID twice daily, cGvHD chronic graft versus host disease.

Requests were available for 1 193 patients, of which data for 1 180 patients were included in the final analysis, the remaining 13 were cancelled requests. In total, 2 541 ruxolitinib requests were included in the analysis (Fig. 1).

### Patient demographics and treatment history

Table 2 shows the demographics and treatment history for the 1 180 patients included in the analysis. Two thirds of patients had cGvHD, over half of whom had severe disease. Of those patients with aGvHD, grade III or IV disease was reported in more than two thirds. In addition, for two thirds of patients there was skin and lower gastrointestinal involvement. Patients were heavily pretreated; >80% had previously received corticosteroids, and 5–8% had previously received ruxolitinib, whereas approximately half of patients with aGvHD had received cyclosporine. Ruxolitinib was predominantly a second- or third-line option for treatment.

**Table 2:** Demographics, disease characteristics and treatment history in patients with cGvHD and aGvHD included in the ruxolitinib CU program analysis.

	Total analysis population	cGvHD	aGvHD
Patients, n (%^a^)	1 180^b^	775 (65.6)	370 (31.4)
Resupply requests, n (%^a^)	498 (42.2)	382 (49.3)	104 (28.1)
Age (years)			
Median (Q1, Q3)	48 (30, 61)	49 (31, 61)	45 (26, 60)
Mean (SD)	46 (61)	45 (19)	42 (20)
Age group (years), n (%^a^)			
2–5	19 (1.6)	12 (1.6)	7 (1.9)
6–11	54 (4.6)	31 (4.0)	22 (6.0)
12–17	41 (3.5)	25 (3.2)	15 (4.1)
≥18	1 067 (90.4)	707 (91.2)	326 (88.1)
Total	1 181^c^ (100.0)	775 (65.7)	370 (31.4)
Gender, n (%^a^)			
Female	490 (41.5)	336 (43.4)	144 (38.9)
Male	665 (56.4)	426 (54.9)	221 (59.7)
Unknown	26 (2.2)	13 (1.7)	5 (1.4)
cGvHD grade, n (%^a^)			
Mild	NA	24 (3.1)	NA
Moderate	NA	305 (39.4)	NA
Severe	NA	431 (55.6)	NA
aGvHD grade, n (%^a^)			
Grade 0	NA	NA	1 (0.3)
Grade I	NA	NA	9 (2.4)
Grade II	NA	NA	102 (27.6)
Grade III	NA	NA	180 (48.6)
Grade IV	NA	NA	71 (19.2)
aGvHD organ involvement, n (%)^a,d^			
Skin	NA	NA	254 (68.6)
Lower GI	NA	NA	252 (68.1)
Upper GI	NA	NA	145 (39.2)
Liver	NA	NA	122 (33.0)
Line of therapy, n (%^a^)			
First	6 (0.5)	3 (0.4)	2 (0.5)
Second	335 (28.4)	163 (21.0)	167 (45.1)
Third	463 (39.2)	327 (42.2)	125 (33.8)
Fourth	206 (17.5)	150 (19.4)	53 (14.3)
>Fourth	136 (11.5)	118 (15.2)	16 (4.3)
Prior treatments^d^			
Corticosteroids	982 (83.2)	628 (81.0)	335 (90.5)
MMF	307 (26.0)	214 (27.6)	85 (23.0)
Cyclosporine	473 (33.9)	292 (37.7)	177 (47.8)
Tacrolimus	272 (23.1)	178 (23.0)	84 (22.7)
ECP	135 (11.4)	104 (13.4)	31 (8.4)
Ruxolitinib	67 (5.7)	35 (4.5)	30 (8.1)

*aGvHD* acute graft versus host disease, *cGvHD* chronic graft versus host disease, *ECP* extracorporeal photopheresis, *GI* gastrointestinal, *MMF* mycophenolate mofetil, *n* number, *NA* not applicable, *Q* quartile, *SD* standard deviation.

^a^Percentages are rounded off, therefore, may not total 100%. Some patient data are missing/incomplete for some baseline variables.

^b^35 patients (3%) reported with other types or non-specified GvHD.

^c^One patient was classed as NA and not included in the final overall population.

^d^Not mutually exclusive.

Canada, Belgium, Australia, Taiwan, Brazil, Korea, Poland, and Israel accounted for 90% of ruxolitinib requests; of these, 32–53% of initial requests resulted in resupplies, with fewer requests for patients with aGvHD (5–36%) than cGvHD (35–62%) (Supplementary Fig. 1). Overall, ruxolitinib resupplies were requested for 42% of patients with an average number of 2.2 supplies (2.4 for cGvHD and 1.7 for aGvHD), equivalent to 198 days of follow-up. The estimated mean (SD; range) duration of ruxolitinib treatment was 176 (150; range 1–676) days for aGvHD and 244 (164; range 2–938) days for cGvHD.

### Ruxolitinib dosage patterns

**Fig. 2:**
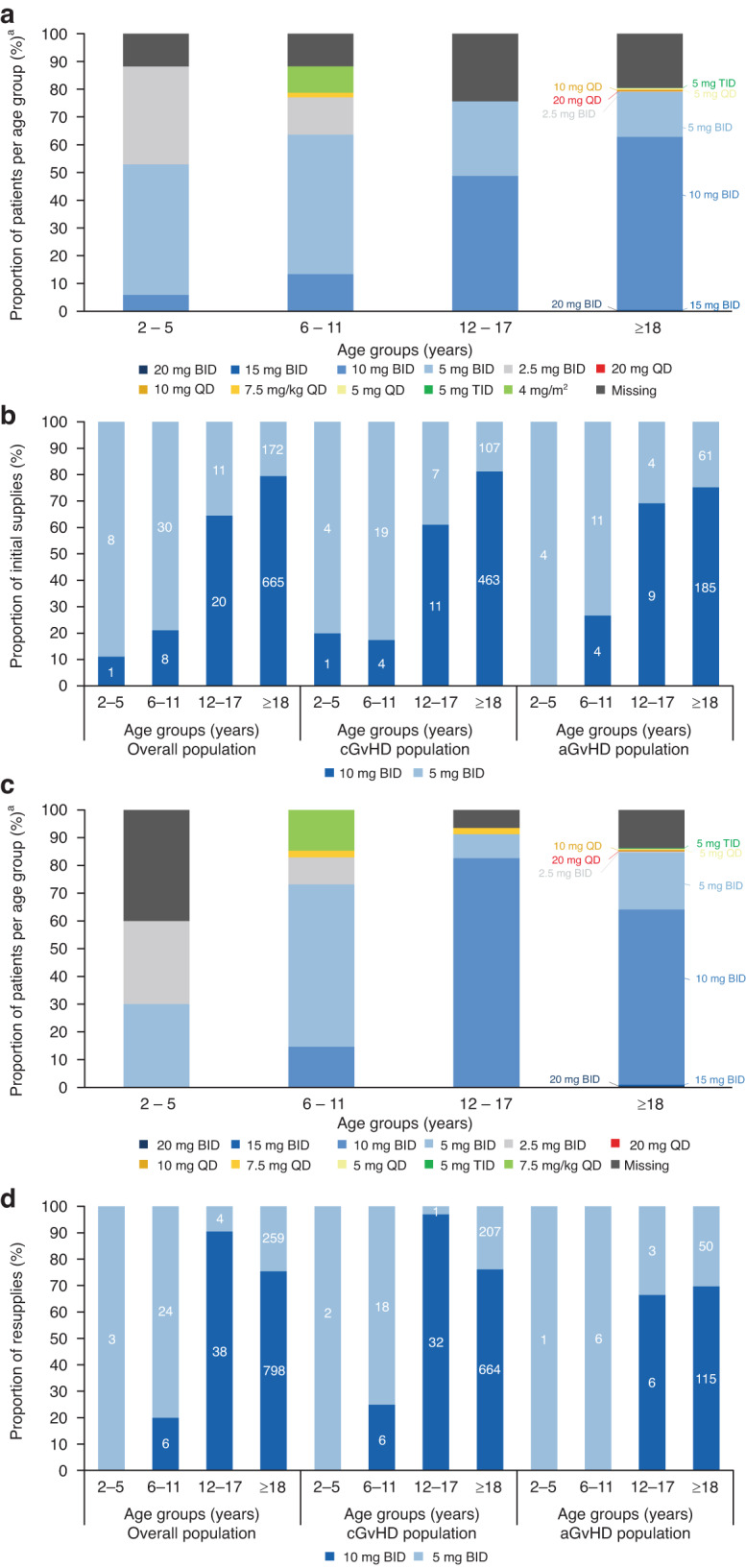
Ruxolitinib doses by age group. Ruxolitinib doses given by physicians^a^ categorized by age for the (**a**) Initial ruxolitinib request (overall population), (**b**) Initial ruxolitinib request in patients with cGvHD and aGvHD (5 mg/10 mg BID population), (**c**) Resupply requests (overall population),^b^ and (**d**) Resupply requests in patients with cGvHD and aGvHD (5 mg/10 mg BID population).^b a^Data were missing or difficult to interpret for 19% and 13% of patients on the initial ruxolitinib request (**b**) and resupply requests (**d**), respectively. ^b^One resupply equates to 3 months of ruxolitinib treatment: data for all resupply requests shown. Data are plotted as a percentage of the overall population (**a**, **c**), or represent patients who received the recommended doses of 5 mg BID or 10 mg BID as a percentage of the total number of patients who received these doses only (**b**, **d**). Patient numbers are shown within the bars. Note: the 10 mg BID dose in young patients (2–11 years) may have been misrepresented due to subjective interpretation of the free text fields in supply/resupply request forms. aGvHD acute graft versus host disease, BID twice daily, cGvHD chronic graft versus host disease.

Both pediatric (range 2–17 years old) and adult ( ≥ 18 years old) patients with aGvHD and cGvHD were treated with a range of ruxolitinib dosing regimens (Fig. 2a, c). The CU treatment plan recommended 5 mg BID and 10 mg BID ruxolitinib for patients ≥6 to <12 years of age and ≥12 years of age, respectively. In general, 5 mg BID and 10 mg BID were the most common ruxolitinib doses used for patients <12 years old and ≥12 years old, respectively, at initial request; however, of patients who received these doses, 5 mg BID was requested for 21% of patients ≥12 years old, rather than the CU recommended dose of 10 mg BID for this age group (Fig. 2b). Notably, the proportion of patients who received 5 mg vs 10 mg BID doses appeared to remain consistent in adults on resupply, whereas the number of teenagers (12–17 years old) with cGvHD who received 10 mg BID ruxolitinib appeared to increase on resupply compared with the initial request (Fig. 2d). For patients aged ≥12 years old who received 5 mg BID or 10 mg BID on resupply, the lower dose of 5 mg BID ruxolitinib was requested for 23% and 30% of patients with cGvHD and aGvHD, respectively.

In the overall disease populations, the proportions of resupply requests for 5 mg BID and 10 mg BID ruxolitinib were similar for patients with aGvHD (25% and 50%, respectively) and cGvHD (21% and 64%, respectively). Dosing regimens other than 5 mg BID or 10 mg BID were used at resupply and data on resupply doses were missing for 13% and 17% of patients with cGvHD and aGvHD, respectively. In general, the overall proportions of patients <12 and ≥12 years old who received 5 mg and 10 mg BID ruxolitinib appeared relatively stable over the first four to six supplies of ruxolitinib (Supplementary Fig. 2).

### Concomitant medication patterns

**Fig. 3:**
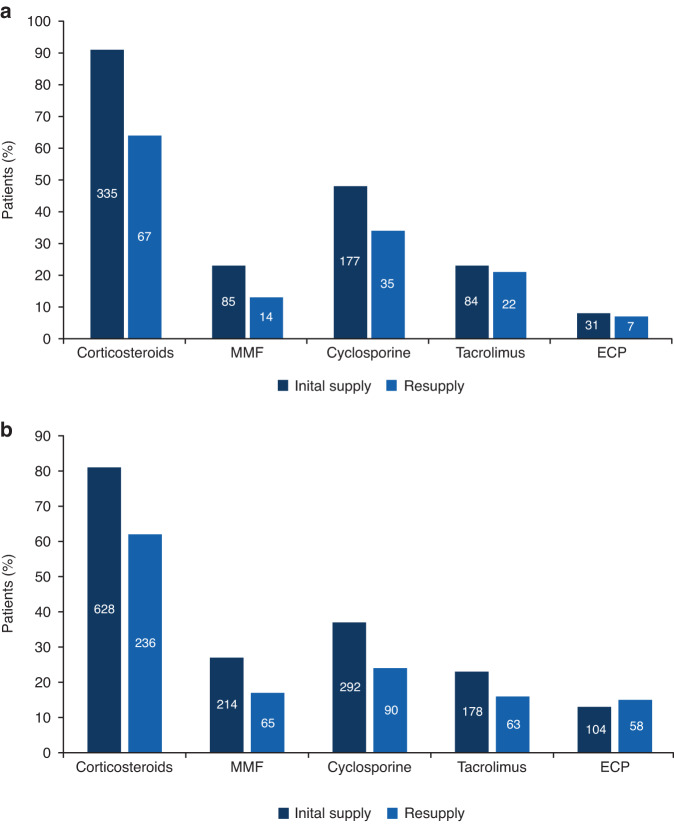
Concomitant medications used at the time of inital supply and resupply of ruxolitinib. Change in concomitant medications used at initial supply request^a^ and after ruxolitinib treatment^b^ in patients treated for (**a**) aGvHD and (**b**) cGvHD. ^a^Data taken at the initial ruxolitinib supply request (before ruxolitinib treatment); ^b^Data shown for concomitant medications over all ruxolitinib resupplies requested. Data represents the percentage of patients/requests in the total aGvHD (*n* = 370) and cGvHD (*n* = 775) populations for initial request data and percentage of resupply requests out of the total number of resupplies for aGvHD (*n* = 104) and cGvHD (*n* = 182) populations. aGvHD acute graft versus host disease, cGvHD chronic graft versus host disease, ECP extracorporeal photopheresis, MMF mycophenolate mofetil.

**Fig. 4:**
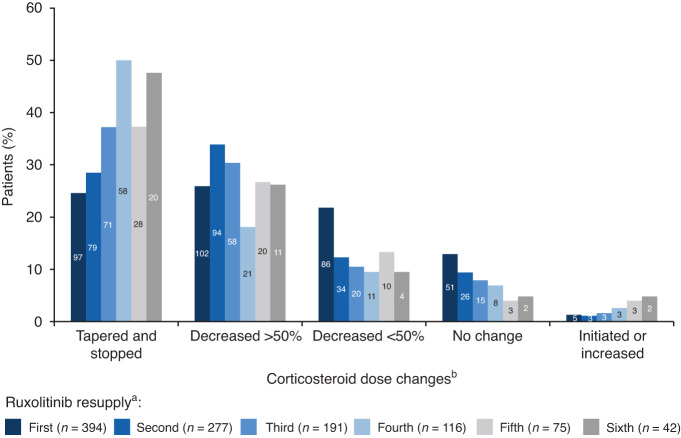
Changes in corticosteroid dose and usage in patients with cGvHD with increasing ruxolitinib resupply number. ^a^These data show the corticosteroid changes between supplies; the corticosteroid dose at initial supply provided the baseline for change in corticosteroid dose at first resupply. ^b^Corticosteroid dose changes in patients at each resupply compared to the previous supply: corticosteroid dose decreased by <50% and patient continued corticosteroids, corticosteroid dose decreased by >50% and patient continued corticosteroids, corticosteroid dose unchanged after initiation of ruxolitinib, corticosteroid dose tapered off and stopped after initiation of ruxolitinib, and corticosteroids initiated or dose increased after initiation of ruxolitinib. Data represents the number of patients with corticosteroid dose changes at each resupply given as a % of all patients at that resupply. Numbers in the bars represent the number of patients. cGvHD chronic graft versus host disease.

Corticosteroid usage was substantially reduced in patients who received ruxolitinib (Fig. 3). Overall, 81% and 91% of patients with cGvHD and aGvHD, respectively, were taking corticosteroids at the time of the initial request for ruxolitinib (Table 1); during ruxolitinib treatment this decreased to 62% and 64%, respectively. A reduction in mycophenolate mofetil and cyclosporine use was also noted. In patients with cGvHD, corticosteroid use was tapered off completely in 26% of patients and 57% could reduce their corticosteroid usage (corticosteroid dose was reduced by >50% in 31% and by <50% in 26%). Fig. 4 shows the changes in corticosteroid dose at each ruxolitinib resupply for patients with cGvHD.

### Response to ruxolitinib treatment

**Fig. 5:**
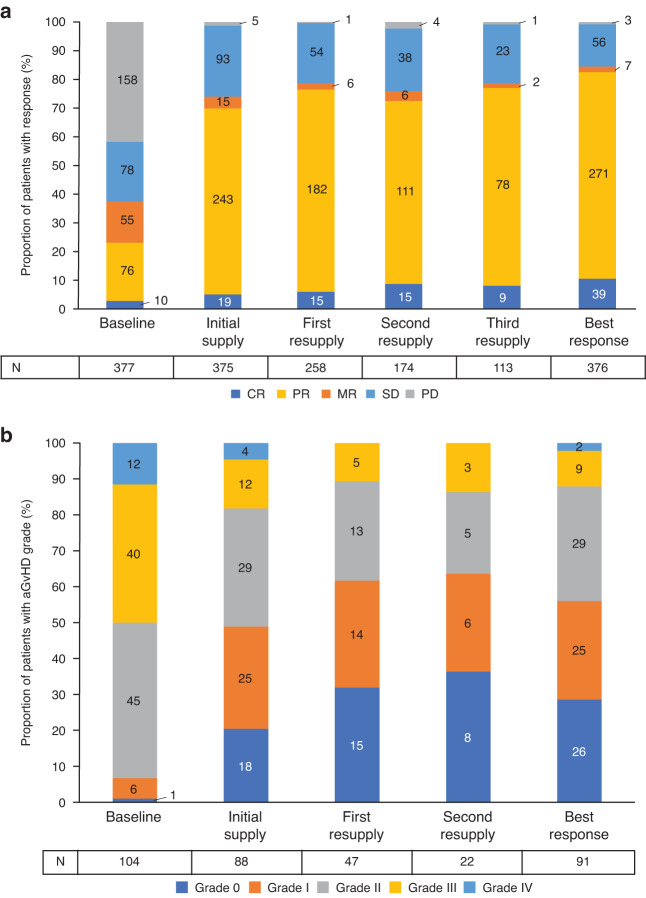
Response to ruxolitinib treatment. Response to ruxolitinib treatment by resupply number and best response in patients with (**a**) cGvHD and (**b**) aGvHD. Data represent the percentage of patients with each grade within the total population at each supply. Baseline indicates the grades captured before the patients received ruxolitinib (at initial request stage) and who later received the initial supply of ruxolitinib and at least one resupply. Initial supply and resupply bars represent the grades captured at the end of these supply periods when physicians were applying for the next ruxolitinib supply. Numbers in bars represent the patient numbers for each response category with total patient numbers for each supply shown in the table. aGvHD acute graft versus host disease, cGvHD chronic graft versus host disease, CR complete response (complete response in all organs), MR mixed response (improvement in at least one organ plus worsening in at least one organ), PD progressive disease (progression in at least one organ); PR partial response (partial response in at least one organ, no progression in others), SD stable disease (no change).

A shift from progressive disease (PD) at baseline (42%) towards complete response (CR) and partial response (PR) was apparent in patients with cGvHD who received ruxolitinib: after the initial supply, 65% of patients had PR and 5% had CR (PD reduced to 1%), which was maintained at least in those patients with resupply (Fig. 5a). Overall, 82% had a best response (BR) of CR or PR (10% and 72%, respectively), with stable disease (SD) in 15% of patients. Notably, 91 (35%) patients reported their BR for at least two consecutive ruxolitinib supplies, 49 (28%) patients for three, and 29 (26%) for four. Adults with cGvHD who received 5 mg BID and 10 mg BID had comparable responses to treatment (CR/PR achieved in 79% and 85%, respectively, with SD in 20.9% vs 12.0%, respectively).

For patients with aGvHD who received ruxolitinib resupplies, grade III/IV aGvHD was reported in 50% of patients at baseline (before ruxolitinib), then 19% and 11% at the end of the initial ruxolitinib supply and first resupply, respectively; overall, 56% of patients had a BR of grade 0/I (Fig. 5b). However, resupplies were received by only 28% of the patients with aGvHD at baseline.

### Safety

Only 11% (124/1 180) of all ruxolitinib requests provided details of AEs; typical AEs included hematological events (anemia, thrombocytopenia, neutropenia), gastrointestinal events (diarrhea), infections (pneumonia), and others, such as hypertension, pyrexia, cough, and fatigue.

## Discussion

CU programs are extensive sources of patient information for investigating new therapies for GvHD in a real-world clinical setting [27–29]. This study is the first analysis of real-world data from the global ruxolitinib CU program for patients with corticosteroid-refractory GvHD.

As expected, the patient population in this global ruxolitinib CU program was heterogenous and heavily pretreated, including pediatric patients <12 years old, predominantly with cGvHD and often with severe disease, which is consistent with results from other real-world studies with ruxolitinib [29, 30] and a US registry [31]. The REACH studies also investigated patients with late stage and severe disease (64% stage III–IV aGvHD; 57% had severe cGvHD) [14, 15] but the inclusion of pediatric patients (from 2 years old) and the number of patients treated in this CU program, and in other ruxolitinib real-world studies [29–31] have greatly expanded the ruxolitinib-treated population beyond the REACH trial populations ( ≥ 12 years old) [14, 15]. Consistent with other CU programs [28–30, 32] many patients in this study had received multiple lines of therapy and concomitant medications, which highlights the complexity of their disease. Interestingly, ruxolitinib was considered predominantly as a second- or third-line treatment option, which suggests that physicians’ have confidence in the efficacy of ruxolitinib versus investigators’ choice of treatment as demonstrated in patients with steroid-refractory aGvHD and cGvHD in the REACH studies, and its safety in vulnerable patients [14, 15].

Notably, most patients received the recommended doses of 5 mg BID and 10 mg BID ruxolitinib ( < 12 years old and ≥12 years old, respectively); around a fifth of adults and patients aged ≥12 years old, however, received the lower dose, 5 mg BID. Many patients would have been treated before the approval of ruxolitinib [33, 34] and physicians would likely exercise discretion and caution when dosing, considering the individual patient’s needs and circumstances, including concomitant medications and underlying conditions; this may, in part, explain the lower ruxolitinib doses used in some patients aged ≥12 years old as well as other ruxolitinib dosing schedules used by physicians. In addition, a reduced dose of ruxolitinib (5 mg BID) is recommended when patients have severe cytopenia, thrombocytopenia, neutropenia, or elevated total bilirubin, or when administered with strong CYP3A4 inhibitors [12, 13]. In other real-world studies of patients with GvHD, a low initial dose of ruxolitinib (5 mg BID) was used in 48–66% of adults in clinical practice, which was subsequently increased to 10 mg BID [32, 35]. A dose of 5 mg once daily was used in 18% of patients with aGvHD and 3% of patients with cGvHD in a real-world safety study [29], whereas tapering of ruxolitinib dose has been reported in 15–38% of patients in other CU studies [28, 30, 35].

Importantly, the tapering off or reduction in corticosteroid dosage was possible in over 83% of patients receiving ruxolitinib. Corticosteroid doses were not captured in this study and, therefore, it was not possible to determine a decrease in the overall corticosteroid exposure. However, the tapering off and reductions in corticosteroid doses and usage reported by physicians suggest a steroid-sparing effect with ruxolitinib. Similarly, in other real-world studies with ruxolitinib, corticosteroid dose was reduced in over 60% of adult and pediatric patients with GvHD [30], whereas at least 75% of patients with cGvHD tapered or discontinued corticosteroids [28, 32]; meanwhile, corticosteroid dose was significantly reduced to a tenth of its original dose after 6 months in patients with aGvHD and to nearly a quarter of the original dose after 12 months in patients with cGvHD (*p* < 0.001) [32, 35]. Collectively, observations from these real-world studies add to the evidence supporting the steroid-sparing effect observed in patients with steroid-refractory/dependent GvHD in REACH2 and REACH3, where patients had consistent reductions in corticosteroid dose over time and more patients discontinued corticosteroids than patients receiving BAT [14, 15].

Lack of response to corticosteroids and, particularly, GvHD disease severity have been linked to poor QoL [4, 5]. In addition to the corticosteroid reductions observed, in those patients who had resupplies, patients responded rapidly to ruxolitinib. Marked reductions in the proportions of patients with stage III/IV aGvHD, and in progressive disease for patients with cGvHD, were evident after the initial dose and maintained by a quarter of patients with cGvHD for up to four ruxolitinib supplies (12 months). This rapid, positive response has also been observed in smaller ruxolitinib real-world and case studies [28, 30, 36, 37] including ORRs of up to 86% in adults and 100% in pediatric patients with GvHD reported within the first 1–2 months of ruxolitinib treatment [30, 37]; similar results have been reported in a small study in patients with steroid-refractory aGvHD who received the monoclonal antibody begelomab within a clinical trial or CU program [38]. The prolonged response to ruxolitinib in our study is consistent with that observed in a retrospective analysis of patients with cGvHD receiving CU ruxolitinib (48.6% and 48.5% after 3 and 12 months of treatment with ruxolitinib) [32]. This retrospective study also highlighted that patients with severe disease had a lower ORR than those with mild/moderate disease (ORR 37.5% and 77.5% in patients with severe vs moderate/mild disease), which supports outcomes from a recent meta-analysis of non-randomized ruxolitinib trials that suggested a possible association between less severe GvHD disease and a better clinical response [32, 39]; however, in our study there were marked reductions in the numbers of patients with grade III/IV aGvHD following the initial ruxolitinib doses. Encouragingly, the response outcomes from the ruxolitinib CU program support the efficacy observed in the REACH2 (ORR 62% at day 28) and REACH3 trials (ORR 49.7% at week 24), which also demonstrated significantly longer failure-free survival and improvements in the Modified Lee Symptom Scale with ruxolitinib compared to investigators’ choice [14, 15, 40]. Likewise, overall and failure-free survival benefits have been observed with ruxolitinib in real-world studies [28, 29, 32, 37] which have been linked to response rates [28, 37]. Although neither survival nor QoL were monitored in our study, collectively, the length of ruxolitinib treatment (up to 12 supplies) and the maintenance of CR/PR over at least four ruxolitinib supplies (12 months) suggest long-term benefits for some patients in this CU program. Ruxolitinib is currently being investigated in treatment-naïve and steroid-refractory pediatric patients ( ≥ 2 years to <18 years old) in the ongoing REACH4 (NCT03491215) [19, 20] and REACH5 (NCT03774082) [21–23] trials, and the doses recommended for pediatric patients in this CU program are consistent with the doses used in these trials. Initial findings suggest a rapid and durable response to ruxolitinib, with ORRs of 91% at day 28 and 69% at day 56 in pediatric patients ≥2 years old with steroid-refractory aGvHD [19]; notably, response rates in pediatric and adult patients with steroid-refractory GvHD were found to be comparable in a meta-analysis of ruxolitinib [41].

Real-world studies are a complementary source of efficacy and safety evidence to that already demonstrated under the strict and selective conditions of randomized controlled trials. These studies are often conducted in larger and more diverse patient populations, reflecting the complexities of clinical care beyond the controlled environment of a clinical trial [42]. However, analysis of real-world data has its limitations. In this analysis, there was a risk of duplicate entries for the same patient where substantial time lapses occurred between resupply requests, and patients who progressed from aGvHD to cGvHD were captured in the aGvHD cohort only. The use of some free text/open fields e.g., for dose, and manual evaluation of supporting documents for these fields, potentially caused some standardization issues, as well as missing data, such as treatment start and end dates, and lack of follow-up on patients not requiring resupplies including the reasons for discontinuation, and details of the corticosteroid doses used. Ruxolitinib was provided as 3-monthy supplies and patient level data were captured every three months at the time of the physician’s resupply request during the near 4-year study period. As a result, endpoints shorter than three months, such as Day 28 response for aGvHD, were not possible with these CU data. In addition, patients had the option to discontinue at any point and, as expected, requests for ruxolitinib resupplies declined over time which inevitably contributed to low patient numbers in some groups, such as those receiving ruxolitinib long-term. The reasons for patient discontinuations from the program were not captured, therefore, the impact of treatment remains unknown in these patients. Analysis was, therefore, limited to areas where the data was largely available and intact, such as patient characteristics, treatment response, dose modifications, ruxolitinib doses used, and treatment patterns. Importantly, the formulaic and complementary baseline and follow-up questions, based on key endpoints in the REACH studies [14, 15] ensured that data was collected consistently from all physicians at each supply stage, providing continuity and standardization for the majority of the information. As a real-world study, potential confounding issues include patients taking undisclosed concomitant medications, unknown compliance with ruxolitinib dosing instructions, and patients being ‘lost to follow-up’, but some of these data may be difficult for the physician to capture and track in the clinic. In addition, we appreciate that there is an inherent bias towards gathering information only in patients assessed as deriving benefit from ruxolitinib and who had product resupply requests; however, standardized information collected over time in these patients will inform on how patients are responding to treatment and how key efficacy parameters may evolve in the real-world setting. Safety data from this CU program was limited but appeared consistent with the safety profile of ruxolitinib [14, 15, 18] and other second/third line options used in GvHD [43]. AEs appeared to be underreported in the current CU program (11% of patients) compared with a real-world study designed to investigate the safety of ruxolitinib in GvHD (68% and 34% of patients with aGvHD and cGvHD had serious AEs, respectively) and, therefore, no clear safety conclusions can be drawn from this CU program; however, the real-world safety study reported no new/unexpected serious AEs and an overall safety profile that was consistent with the REACH trials [29].

In summary, CU programs are a valuable source of additional real world clinical data that can validate and expand on the outcomes from clinical trials, highlighting patterns in prescribing behavior and patient responses. In this ruxolitinib CU program, the reductions in corticosteroid use in addition to substantial and durable reductions in disease severity highlight that patients responded well to ruxolitinib, even after the first supply, and supports the efficacy of ruxolitinib observed in Phase III trials.

### Supplementary information

Supplementary Material and Figures

Supplementary Table 1. CU program criteria and medical inclusion criteria for patients

Supplementary Table 2. Overview of the full questions and answers, including drop-down options, posed to physicians at baseline and ruxolitinib resupply for patients with aGvHD and cGvHD

## Data Availability

Individual data sharing to third parties will not be possible. Access to aggregated data might be granted following review. Such requests can be submitted to the corresponding author for consideration.

## References

[1] Zeiser R, Blazar BR (2017). Acute Graft-versus-Host Disease - Biologic Process, Prevention, and Therapy. N. Engl J Med.

[2] Lee SJ, Kim HT, Ho VT, Cutler C, Alyea EP, Soiffer RJ (2006). Quality of life associated with acute and chronic graft-versus-host disease. Bone Marrow Transplant.

[3] Kurosawa S, Oshima K, Yamaguchi T, Yanagisawa A, Fukuda T, Kanamori H (2017). Quality of Life after Allogeneic Hematopoietic Cell Transplantation According to Affected Organ and Severity of Chronic Graft-versus-Host Disease. Biol Blood Marrow Transplant.

[4] Hamad N, Lachance S, De Courcy J, Rowaichi L, Zuurman M, Mohty M (2021). Patient-Reported Outcomes in Acute Graft-Vs-Host Disease: Quality-Of-Life Findings from a Real-World Study. Bone Marrow Transplant.

[5] Lachance S, Hamad N, De Courcy J, Gibson G, Zuurman M, Mohty M (2021). Impact of Chronic Gvhd Severity and Steroid Response on the Quality of Life in Patients Following Allogeneic Stem Cell Transplantation: Findings from A Real-World Study. Bone Marrow Transplant.

[6] Martin PJ, Rizzo JD, Wingard JR, Ballen K, Curtin PT, Cutler C (2012). First- and second-line systemic treatment of acute graft-versus-host disease: recommendations of the American Society of Blood and Marrow Transplantation. Biol Blood Marrow Transplant.

[7] Ruutu T, Gratwohl A, de Witte T, Afanasyev B, Apperley J, Bacigalupo A (2014). Prophylaxis and treatment of GVHD: EBMT-ELN working group recommendations for a standardized practice. Bone Marrow Transplant.

[8] Penack O, Marchetti M, Ruutu T, Aljurf M, Bacigalupo A, Bonifazi F (2020). Prophylaxis and management of graft versus host disease after stem-cell transplantation for haematological malignancies: updated consensus recommendations of the European Society for Blood and Marrow Transplantation. Lancet Haematol.

[9] MacMillan ML, Weisdorf DJ, Wagner JE, DeFor TE, Burns LJ, Ramsay NK (2002). Response of 443 patients to steroids as primary therapy for acute graft-versus-host disease: comparison of grading systems. Biol Blood Marrow Transplant.

[10] Westin JR, Saliba RM, De Lima M, Alousi A, Hosing C, Qazilbash MH (2011). Steroid-Refractory Acute GVHD: Predictors and Outcomes. Adv Hematol.

[11] Jaglowski SM, Devine SM (2014). Graft-versus-host disease: why have we not made more progress?. Curr Opin Hematol.

[12] Novartis Pharmaceuticals. Jakavi (Ruxolitinib) Summary of Product Characteristics. https://www.ema.europa.eu/en/documents/product-information/jakavi-epar-product-information_en.pdf. Accessed 22 March 2023.

[13] Novartis Pharmaceuticals. Jakavi (Ruxolitinib) Prescribing Information. https://www.jakafi.com/pdf/prescribing-information.pdf. Accessed 22 March 2023.

[14] Zeiser R, von Bubnoff N, Butler J, Mohty M, Niederwieser D, Or R (2020). Ruxolitinib for Glucocorticoid-Refractory Acute Graft-versus-Host Disease. N. Engl J Med.

[15] Zeiser R, Polverelli N, Ram R, Hashmi SK, Chakraverty R, Middeke JM (2021). Ruxolitinib for Glucocorticoid-Refractory Chronic Graft-versus-Host Disease. N. Engl J Med.

[16] Aliu P, Sarp S, Fitzsimmons P (2021). Increasing Use of Compassionate Use/Managed Access Channels to Obtain Medicines for Use in COVID-19. Clin Pharmacol Ther.

[17] Aliu P, Sarp S, Reichenbach R, Behr S, Fitzsimmons P, Shamlajee M (2022). International Country-Level Trends, Factors, and Disparities in Compassionate Use Access to Unlicensed Products for Patients With Serious Medical Conditions. JAMA Health Forum.

[18] Jagasia M, Perales MA, Schroeder MA, Ali H, Shah NN, Chen YB (2020). Ruxolitinib for the treatment of steroid-refractory acute GVHD (REACH1): a multicenter, open-label phase 2 trial. Blood.

[19] Locatelli F, Kang HJ, Bruno B, Gandemer V, Rialland F, Faraci M (2022). Ruxolitinib in Pediatric Patients with Treatment-Naïve or Steroid-Refractory Acute Graft-Versus-Host Disease: Primary Findings from the Phase I/II REACH4 Study. Blood.

[20] ClinicalTrials.gov. Study of pharmacokinetics, activity and safety of ruxolitinib in pediatric patients with grade II-IV acute graft vs. host disease. https://clinicaltrials.gov/ct2/show/NCT03491215. Accessed 22 March 2023.

[21] Wall D, Koh K, Bhat S, Takahashi Y, Zhang A, Rosko C (2021). A Phase 2 Open-label, Single-arm, Multicenter Study of Ruxolitinib Added to Corticosteroids in Pediatric Subjects with Moderate/Severe Chronic Gvhd after Allogeneic Stem Cell Transplantation (REACH5). Bone Marrow Transplant.

[22] ClinicalTrials.gov. Activity, safety and pharmacokinetics in pediatric subjects with moderate and severe chronic graft vs. host disease after allogeneic stem cell transplant. https://clinicaltrials.gov/ct2/show/NCT03774082?term=NCT03774082&draw=2&rank=1. Accessed 22 March 2023.

[23] Locatelli F, Antmen B, Kang HJ, Koh K, Takahashi Y, Kupesiz A (2023). Ruxolitinib in pediatric patients with treatment-naïve or steroid-refractory chronic graft versus host disease: primary findings from the phase II REACH5 study. Hemasphere.

[24] Lee SJ, Wolff D, Kitko C, Koreth J, Inamoto Y, Jagasia M (2015). Measuring therapeutic response in chronic graft-versus-host disease. National Institutes of Health consensus development project on criteria for clinical trials in chronic graft-versus-host disease: IV. The 2014 Response Criteria Working Group report. Biol Blood Marrow Transpl.

[25] Harris AC, Young R, Devine S, Hogan WJ, Ayuk F, Bunworasate U (2016). International, Multicenter Standardization of Acute Graft-versus-Host Disease Clinical Data Collection: A Report from the Mount Sinai Acute GVHD International Consortium. Biol Blood Marrow Transpl.

[26] Jagasia MH, Greinix HT, Arora M, Williams KM, Wolff D, Cowen EW (2015). National Institutes of Health Consensus Development Project on Criteria for Clinical Trials in Chronic Graft-versus-Host Disease: I. The 2014 Diagnosis and Staging Working Group report. Biol Blood Marrow Transpl.

[27] Macías-Sánchez MDM, Morata-Tarifa C, Cuende N, Cardesa-Gil A, Cuesta-Casas M, Pascual-Cascon MJ (2022). Mesenchymal Stromal Cells for Treating Steroid-Resistant Acute and Chronic Graft Versus Host Disease: A Multicenter Compassionate Use Experience. Stem Cells Transl Med.

[28] Redondo S, Esquirol A, Novelli S, Caballero AC, Garrido A, Oñate G (2022). Efficacy and Safety of Ruxolitinib in Steroid-Refractory/Dependent Chronic Graft-versus-Host Disease: Real-World Data and Challenges. Transplant Cell Ther.

[29] Schroeder MA, Hari PN, Blithe A, Paranagama D, Bhatt V, DiPersio JF (2022). Safety analysis of patients who received ruxolitinib for steroid-refractory acute or chronic graft-versus-host disease in an expanded access program. Bone Marrow Transplant.

[30] Escamilla Gomez V, García Gutiérrez V, Astibia Mahillo B, Alcalde P, López Corral L, Acera Gómez M (2022). Ruxolitinib in Acute and Chronic Graft-Versus-Host Disease: Real Life Experience in a Multi-Centre Study. Blood.

[31] Ingram A, Shaw BE, Covill S, Kaur M, Huang X (2022). Analysis of US Registry Data on Patient Characteristics, Treatment Patterns and Outcomes of Patients Receiving Extracorporeal Photopheresis with or without Ruxolitinib. Blood.

[32] White J, Elemary M, Linn SM, Novitzky-Basso I, Culos S, Tan SK (2023). A Multicenter, Retrospective Study Evaluating Clinical Outcomes of Ruxolitinib Therapy In Heavily Pretreated Chronic GVHD Patients With Steroid Failure. Transplant Cell Ther.

[33] Food and Drug Administration. FDA approves ruxolitinib for acute graft-versus-host disease. https://www.fda.gov/drugs/resources-information-approved-drugs/fda-approves-ruxolitinib-acute-graft-versus-host-disease. Accessed 22 March 2023.

[34] Food and Drug Administration. FDA approves ruxolitinib for chronic graft-versus-host disease. https://www.fda.gov/drugs/resources-information-approved-drugs/fda-approves-ruxolitinib-chronic-graft-versus-host-disease. Accessed 22 March 2023.

[35] Murray A, Linn SM, Yu B, Novitzky-Basso I, Mattsson J, Elemary M (2022). Real-World Experience with Ruxolitinib Therapy for Steroid Refractory Acute Graft Versus Host Disease. Blood.

[36] Sarmiento Maldonado M, Ramírez Villanueva P, Bertín Cortes-Monroy P, Jara Arias V, Soto Donoso K, Uribe Gonzalez P (2017). Compassionate use of ruxolitinib in acute and chronic graft versus host disease refractory both to corticosteroids and extracorporeal photopheresis. Exp Hematol Oncol.

[37] Leung GMK, Sim JPY, Hwang YY, Chan TSY, Lie AKW, Tse E (2022). Ruxolitinib in the management of steroid-resistant/-dependent acute and chronic graft-versus-host disease: results of routine practice in an academic centre. Ann Hematol.

[38] Bacigalupo A, Angelucci E, Raiola AM, Varaldo R, Di Grazia C, Gualandi F (2020). Treatment of steroid resistant acute graft versus host disease with an anti-CD26 monoclonal antibody-Begelomab. Bone Marrow Transplant.

[39] Zhang M-y, Zhao P, Zhang Y, Wang J-s (2022). Efficacy and safety of ruxolitinib for steroid-refractory graft-versus-host disease: Systematic review and meta-analysis of randomised and non-randomised studies. PLOS ONE.

[40] Mohty M, Bulabois C-E, García-Gutiérrez V, Ritchie D, Yoon S-S, Coiteux V (2021). Ruxolitinib (RUX) Vs Best Available Therapy (BAT) in Patients With Steroid-refractory Acute GRAFT-VS HOST Disease (SR-AGVHD): 6-Month Follow-up From The Randomized, Phase 3 Reach2 Study. Bone Marrow Transplant.

[41] Fan S, Huo WX, Yang Y, Shen MZ, Mo XD (2022). Efficacy and safety of ruxolitinib in steroid-refractory graft-versus-host disease: A meta-analysis. Front Immunol.

[42] Azoulay L (2022). Rationale, Strengths, and Limitations of Real-World Evidence in Oncology: A Canadian Review and Perspective. Oncologist.

[43] Velickovic VM, McIlwaine E, Zhang R, Spelman T (2020). Adverse events in second- and third-line treatments for acute and chronic graft-versus-host disease: systematic review. Ther Adv Hematol.

