# Supplementary material for: Ruxolitinib in patients with graft versus host disease (GvHD): findings from a compassionate use program
Source: Bone Marrow Transplant. 2024 Feb 15;59(5):637–46. doi: 10.1038/s41409-024-02207-4 (PMC11073975; doi:10.1038/s41409-024-02207-4)
Supplement: Supplementary file 1 — Supplementary Material and Figures [file 41409_2024_2207_MOESM1_ESM.pdf]

Ruxolitinib dose modifications

To allow patients to continue on the ruxolitinib compassionate use (CU) program, dose adjustments were permitted for those who could not tolerate the dosing schedule specified in the treatment plan, and dose reductions or interruptions for worsening cytopenias or non-hematological toxicity attributed to ruxolitinib.

Dose level	Proposed dose	First dose reduction	Second dose reduction
1	10 mg BID	5 mg BID	5 mg QD
2	5 mg BID	2.5 mg BID	2.5 mg QD
3	4 mg/m^2 BID[a]	2 mg/m^2 BID[a]	2 mg/m^2 QD[a]

[a]BSA scaled dose to be rounded to the nearest 0.5mg

BID, twice daily; BSA, body surface area; QD, once daily

Ruxolitinib dosing can be restarted or increased after recovery of the hematological parameter to acceptable levels such that patients may attain the highest tolerated dosing regimen to obtain a clinical response (one dose level increase allowed every 2 weeks):

Dose level	Reduced dose	First dose escalation	Second dose escalation
1	5 mg QD	5 mg BID	10 mg BID
2	2.5 mg QD	2.5 mg BID	5 mg BID
3	2 mg/m^2 QD[a]	2 mg/m^2 BID[a]	4 mg/m^2 BID[a]

[a]BSA scaled dose to be rounded to the nearest 0.5mg

BID, twice daily; BSA, body surface area; QD, once daily

For patients discontinuing ruxolitinib for reasons other than safety, for example in the event of a complete response to treatment, a tapering strategy may be adopted to avoid graft versus host disease (GvHD) flare up (based on the condition of the patient, current dosing regimen and clinical judgment of the physician):

- 50% dose reduction every 2 months (56 days) i.e. initial dose reduction from 10 mg BID to 5 mg BID
- if GvHD stable disease is sustained, dose is further tapered by a second 50% dosage reduction to 5 mg QD for an additional 56 days, prior to cessation.

Supplementary Table 1. CU program criteria and medical inclusion criteria for patients [See separate file]

Supplementary Table 2. Overview of the full questions and answers, including drop-down options, posed to physicians at baseline and ruxolitinib resupply for patients with aGvHD and cGvHD [See separate file]

Supplementary Table 3. Global safety checks performed based on physicians' responses to follow-up questions at resupply[a]

**Ruxolitinib aGvHD:**<br><br>**Have you been modifying the dose of the CS since initiation of ruxolitinib?**<br><br>If answer is CS initiated or CS dose increased after ruxolitinib then a Global Safety Check is performed.<br><br>Note: if the CS dose increases, there is also a check on the answer to the grades question: What is the overall grade of aGVHD at present?
**Ruxolitinib cGvHD:**<br><br>**Please specify the overall response to ruxolitinib according to NIH Consensus for measuring Therapeutic response[1]**<br><br>If answer is Progression in at least one organ, Global Safety Check is performed.<br><br>• **Have you been modifying the dose of the CS since initiation of ruxolitinib?**<br><br>If answer is CS initiated OR CS dose increased after ruxolitinib, Global Safety Check is performed.<br><br>• **Please provide systemic therapy(ies) used at present to treat cGvHD (in addition to ruxolitinib)**<br><br>If answer is yes to systemic therapies in addition to ruxolitinib, Global Safety Check is performed.

[a]No safety checks performed at baseline (patients not yet in the program). Country-specific safety representatives followed-up on physicians' responses to resupply questions regarding potential adverse events for databasing in the ARGUS safety database.

[1] Lee SJ, et al. *Biol Blood Marrow Transplant*. 2015; 21:984–99

aGvHD, acute graft versus host disease; cGvHD, chronic graft versus host disease; CS, corticosteroid.

Supplementary Table 4. Variables extracted from Novartis online CU system for data analysis

Variables	Operationalization
Age (years)	Difference between first treatment date or request submit date and date of birth
Gender	Male<br>Female
cGvHD grading	Mild, moderate, severe
aGvHD organ involvement	Skin, lower GI, upper GI, liver
Line of therapy	1st, 2nd, 3rd, 4th, >4th
aGvHD grading	0, I, II, III, IV
Ruxolitinib dose	5 mg BID, 10 mg BID, others
Treatment duration	Number
Response during follow-up	Complete response, partial response, stable disease, progressive disease, mixed response
Prior treatments	Corticosteroids, MMF, cyclosporine, tacrolimus, ECP, others[a]

[a]see **Supplementary Table 2** for complete list of prior treatments

aGvHD, acute graft versus host disease; BID, twice daily; cGvHD, chronic graft versus host disease; CS, corticosteroid; CU, compassionate use; ECP, extracorporeal photopheresis; GI, gastrointestinal; MMF, mycophenolate mofetil.

Supplementary Figure 1. Geographic distribution of the initial ruxolitinib requests and resupplies for (A) all patients, and patients with (B) cGvHD and (C) aGvHD

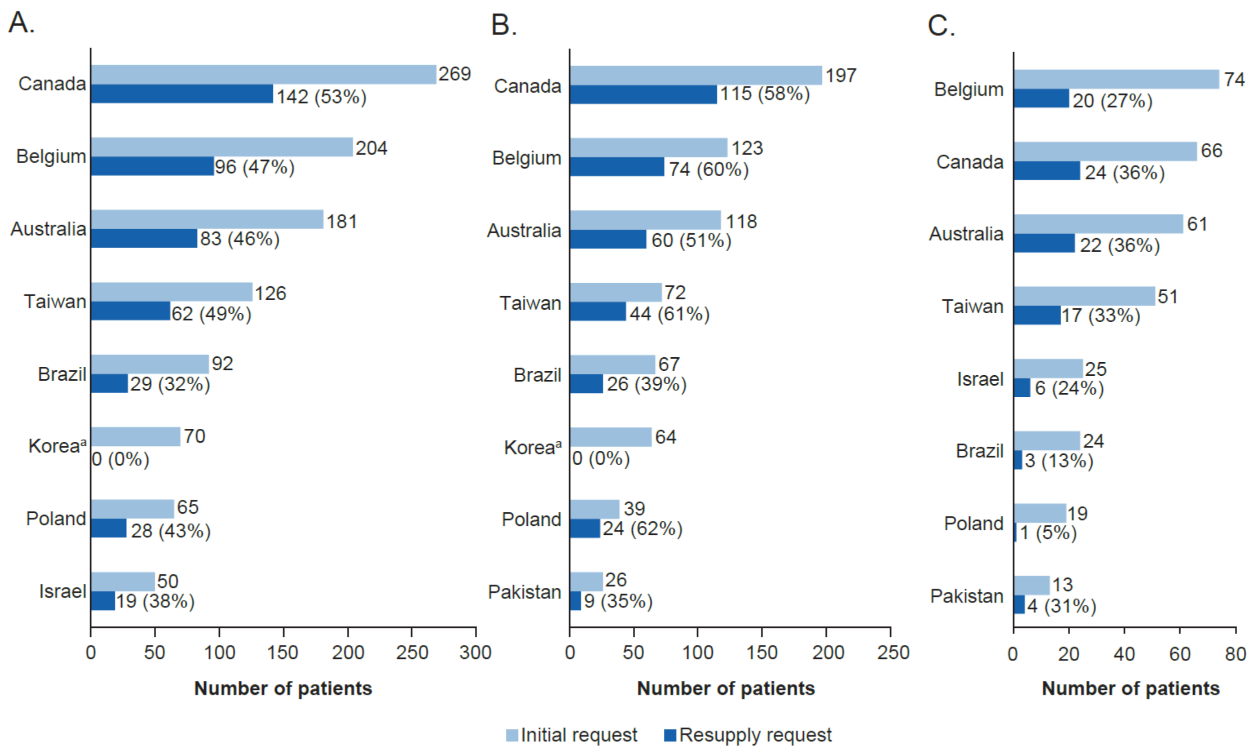

[a]No resupply requests were received from South Korea as regulations do not allow resupplies after the initial country-specific 6-month supply. All other countries received an initial 3-month supply and subsequent 3-month resupplies.

Numbers in parentheses denote the percentage of resupply requests compared to the initial ruxolitinib supply.

aGvHD, acute graft versus host disease; cGvHD, chronic graft versus host disease.

Supplementary Figure 2. Change in ruxolitinib dose by age and by ruxolitinib supply in patients with (A) aGvHD and (B) cGvHD

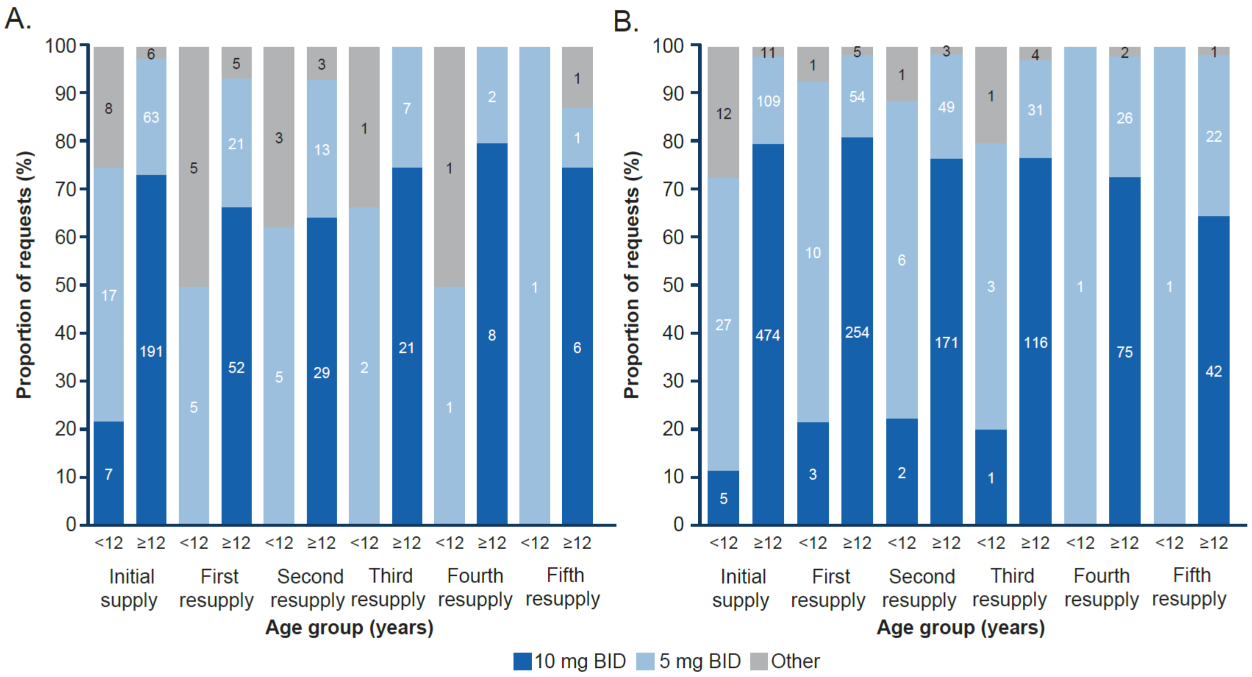

Data represent the % of requests for 10 mg BID, 5 mg BID and all other doses in the <12-year-old population and >12-year-old populations at each ruxolitinib supply. Data in bars are the number of requests at each dose.

aGvHD, acute graft versus host disease; BID, twice daily; cGvHD, chronic graft versus host disease.
