# Supplementary material for: Ruxolitinib in patients with graft versus host disease (GvHD): findings from a compassionate use program
Source: Bone Marrow Transplant. 2024 Feb 15;59(5):637–46. doi: 10.1038/s41409-024-02207-4 (PMC11073975; doi:10.1038/s41409-024-02207-4)
Supplement: Supplementary file 2 — Supplementary Table 1. CU program criteria and medical inclusion criteria for patients [file 41409_2024_2207_MOESM2_ESM.pdf]

Supplementary Table 1. CU program criteria and medical inclusion criteria for patients

**CU program criteria[a]**	
An independent request should be received from the physician or other Health Care Professional (HCP), where regulations allow	
Patient to be treated has a serious or life-threatening disease or condition, and no comparable or satisfactory alternative therapy is available to monitor or treat the disease or condition	
Patient is not eligible or able to enroll in a clinical trial	
There is a potential patient benefit to justify the potential risk of the treatment use, and the potential risk is not unreasonable in the context of the disease or condition to be treated	
The patient must meet any other important medical criteria established by the medical experts working on the product development program (see medical inclusion/exclusion criteria below)	
Provision of the investigational product will not interfere with the initiation, conduct or completion of a Novartis clinical trial or overall development program	
Such access provision as described above is allowed as per local laws and regulations	
**Medical inclusion criteria[b]**	**Medical exclusion criteria[c]**
Male or female patients age ≥2 years	History of hypersensitivity to any drugs or metabolites of similar chemical classes as ruxolitinib
Patients with a confirmed diagnosis of aGvHD or c(SR)-GVHD<br>• Clinically diagnosed grade II to IV aGvHD[1] requiring systemic IST<br>• Clinically diagnosed moderate to severe stage cGvHD[2] requiring systemic IST	Presence of an active uncontrolled infection including significant bacterial, fungal, viral (including CMV, EBV, HHV-6, HBV, HCV, BK virus or HIV) or parasitic infection requiring treatment. Infections are considered controlled if appropriate therapy has been instituted and, at the time of screening, no signs of progression are

	present. Progression of infection is defined as hemodynamic instability attributable to sepsis, new symptoms, worsening physical signs or radiographic findings attributable to infection. Persisting fever without other signs or symptoms will not be interpreted as progressing infection
Evident myeloid and platelet engraftment with ANC >1000/mm^3 and platelets >20,000/mm^3 (GF supplementation and transfusion support allowed)	Evidence of active tuberculosis (clinical diagnosis per local practice)
Written patient informed consent must be obtained prior to start of treatment.	Presence of severely impaired renal function defined by serum creatinine >2 mg/dL (>176.8 µmol/L), or have estimated creatinine clearance <30 mL/min measured or calculated by Cockroft Gault equation or calculated by the updated bedside Schwartz equation, or a serum creatinine greater than specified in the table below based on age/gender: (confirmed within 72h prior to treatment start):<br>Age, years / Max. Serum Creatinine (mg/dL): Male / Female<br>2 to <6: 0.8 / 0.8<br>6 to <10: 1.0 / 1.0<br>10 to 13: 1.2 / 1.2<br>13 to <16: 1.5 / 1.4<br>≥16: 1.7 / 1.4
	Concomitant use of another JAK inhibitor
	Pregnant or nursing (lactating) women

	Not able to understand and to comply with treatment instructions and requirements.
	Women of child-bearing potential, defined as all women physiologically capable of becoming pregnant, unless they are:<br>• women whose sexual orientation precludes intercourse with a male partner<br>• women whose partners have been sterilized by vasectomy or other means<br>• using a highly effective method of birth control (i.e. one that results in a less than 1% per year failure rate when used consistently and correctly); periodic abstinence is not acceptable throughout the period of treatment and 30 days after treatment discontinuation

[a]Criteria must be fulfilled for the provision of agents for CU; [b]Patients eligible for inclusion in the ruxolitinib CU program must meet all the medical inclusion criteria; [c]Patients eligible for the ruxolitinib CU program must not meet any of the exclusion criteria

[1] Harris AC, et al. International, Multicenter Standardization of Acute Graft-versus-Host Disease Clinical Data Collection: A Report from the Mount Sinai Acute GvHD International Consortium. *Biol Blood Marrow Transplant*. 2016 Jan;22(1):4–10.

[2] Jagasia MH, et al. National Institutes of Health Consensus Development Project on Criteria for Clinical Trials in Chronic Graft-versus-Host Disease: I. The 2014 Diagnosis and Staging Working Group report. *Biol Blood Marrow Transplant.* 2015 Mar;21(3):389-401.e1.

aGvHD, acute graft versus host disease; ANC, absolute neutrophil count; cGvHD, chronic graft versus host disease; CMV, cytomegalovirus; CU, compassionate use; EBV, Epstein-Barr virus; GF, growth factor; HBV, hepatitis B virus; HCV, hepatitis C virus; HHV-6, human herpes virus-6; HIV, human immunodeficiency virus; IST, immune suppressive therapy; JAK, Janus kinase; SR, steroid refractory.
