# Supplementary material for: Ruxolitinib in patients with graft versus host disease (GvHD): findings from a compassionate use program
Source: Bone Marrow Transplant. 2024 Feb 15;59(5):637–46. doi: 10.1038/s41409-024-02207-4 (PMC11073975; doi:10.1038/s41409-024-02207-4)
Supplement: Supplementary file 3 — Supplementary Table 2. Overview of the full questions and answers, including drop-down options, posed to physicians at baseline and ruxolitinib resupply for patients with aGvHD and cGvHD [file 41409_2024_2207_MOESM3_ESM.pdf]

Supplementary Table 2. Overview of the full questions and answers, including drop-down options, posed to physicians at baseline and ruxolitinib resupply for patients with aGvHD and cGvHD

**Steroid-refractory aGvHD**	
**Baseline questions**	**Resupply questions**
Is your request for aGvHD?<br>• Yes<br>• No<br>**If yes, the following questions are displayed**	Is your request for aGvHD?<br>• Yes<br>• No<br>**If yes, the following questions are displayed**
Please tick the relevant boxes for organ involvement of aGvHD at present:<br>• Skin (Drop-down menu: Stage 0, Stage 1, Stage 2, Stage 3, Stage 4)<br>• Lower GI (Drop-down menu: Stage 0, Stage 1, Stage 2, Stage 3, Stage 4)<br>• Upper GI (Drop-down menu: Stage 0, Stage 1, Stage 2, Stage 3, Stage 4)<br>• Liver (Drop-down menu: Stage 0, Stage 1, Stage 2, Stage 3, Stage 4)	Please tick the boxes for organ involvement at present:<br>• Skin (Drop-down menu: Stage 0, Stage 1, Stage 2, Stage 3, Stage 4)<br>• Lower GI (Drop-down menu: Stage 0, Stage 1, Stage 2, Stage 3, Stage 4)<br>• Upper GI (Drop-down menu: Stage 0, Stage 1, Stage 2, Stage 3, Stage 4)<br>• Liver (Drop-down menu: Stage 0, Stage 1, Stage 2, Stage 3, Stage 4)
What is the overall grade of aGvHD at present[1]? Drop-down menu:<br>• Grade 0: No stage 1–4 of any organ.<br>• Grade I: Stage 1–2 skin without liver, upper GI, or lower GI involvement<br>• Grade II: Stage 3 rash and/or stage 1 liver and/or stage 1 upper GI and/or stage 1 lower GI<br>• Grade III: Stage 2–3 liver and/or stage 2–3	What is the overall grade of aGvHD at present[1]? Drop-down menu:<br>• Grade 0: No stage 1–4 of any organ.<br>• Grade I: Stage 1–2 skin without liver, upper GI, or lower GI involvement<br>• Grade II: Stage 3 rash and/or stage 1 liver and/or stage 1 upper GI and/or stage 1 lower GI<br>• Grade III: Stage 2–3 liver and/or stage 2–3

lower GI, with stage 0–3 skin and/or stage 0–1 upper GI<br>• Grade IV: Stage 4 skin, liver, or lower GI involvement, with stage 0–1 upper GI	lower GI, with stage 0–3 skin and/or stage 0–1 upper GI<br>• Grade IV: Stage 4 skin, liver, or lower GI involvement, with stage 0–1 upper GI
Which line of treatment is ruxolitinib going to be used for?<br>Drop-down menu:<br>• 1st<br>• 2nd<br>• 3rd<br>• 4th<br>• Beyond 4th line	Have you been modifying the dose of the corticosteroids (CS) since initiation of ruxolitinib?<br>Drop-down menu:<br>• Patient was not on CS prior to ruxolitinib<br>• CS dose tapered off and stopped after initiation of ruxolitinib<br>• CS dose decreased more than 50% and patient still on CS<br>• CS dose decreased less than 50% and patient still on CS<br>• CS dose not changed after initiation of ruxolitinib<br>• CS initiated or CS dose increased after ruxolitinib
Please provide systemic therapy(ies) that are currently in use to treat aGvHD.<br>Multiselect list (multiple options can be selected):<br>• Anti-TNF antibody<br>• ATG<br>• Cyclosporine<br>• Dexamethasone<br>• ECP	Please provide systemic therapy(ies) used at present to treat aGvHD (in addition to ruxolitinib).<br>Multiselect list (multiple options can be selected):<br>• Anti-TNF antibody<br>• ATG<br>• Cyclosporine<br>• Dexamethasone<br>• ECP

• Etanercept<br>• Itacitinib<br>• Mesenchymal stem cells<br>• Methotrexate<br>• Methylprednisolone<br>• Mycophenolate Mofetil<br>• Prednisone<br>• Sirolimus<br>• Tacrolimus<br>• Other JAK inhibitor<br>• Other monoclonal antibody<br>• Other	• Etanercept<br>• Itacitinib<br>• Mesenchymal stem cells<br>• Methotrexate<br>• Methylprednisolone<br>• Mycophenolate Mofetil<br>• Prednisone<br>• Sirolimus<br>• Tacrolimus<br>• Other JAK inhibitor<br>• Other monoclonal antibody<br>• Other
**Steroid-refractory cGvHD**	
**Baseline questions**	**Resupply questions**
Is your request for cGvHD?<br>• Yes<br>• No<br>**If yes, the following questions are displayed**	Is your request for cGvHD?<br>• Yes<br>• No<br>**If yes, the following questions are displayed**
Has the patient presented with acute GvHD prior?<br>• Yes<br>• No	Please specify the overall response to ruxolitinib according to NIH Consensus for measuring Therapeutic response[2].<br>Dropdown menu:<br>• CR: Complete response in all organs<br>• PR: Partial response in at least one organ, no progression in others

	• MR: Improvement in at least one organ plus worsening in at least one organ<br>• SD: No change<br>• PD: Progression in at least one organ
Which line of chronic GvHD treatment is ruxolitinib going to be used at?<br>Dropdown menu:<br>• 1st<br>• 2nd<br>• 3rd<br>• 4th<br>• Beyond 4th line	What is the overall severity of cGvHD at present (NIH)[3]?<br>Dropdown menu:<br>• Mild: 1 or 2 organs involved with no more than score 1 plus lung score 0<br>• Moderate: 3 or more organs involved with no more than score 1 OR at least 1 organ (not lung) with a score of 2 OR lung score 1<br>• Severe: At least 1 organ with a score of 3 OR lung score of 2 or 3<br>• None
Please provide systemic therapy(ies) that are currently in use to treat chronic GvHD.<br>Multiselect list (multiple options can be selected):<br>• ATG<br>• Aldesleukin (IL-2)<br>• Azathioprine<br>• Bortezomib<br>• Cyclosporine<br>• Dexamethasone<br>• ECP<br>• Etanercept	Have you been modifying the dose of the corticosteroids (CS) since initiation of ruxolitinib?<br>Drop-down menu:<br>• Patient was not on CS prior to ruxolitinib<br>• CS dose tapered off and stopped after initiation of ruxolitinib<br>• CS dose decreased more than 50% and patient still on CS<br>• CS dose decreased less than 50% and patient still on CS<br>• CS dose not changed after initiation of ruxolitinib

• Ibrutinib<br>• IL inhibitor<br>• Infliximab<br>• Methotrexate<br>• Methylprednisolone<br>• Mycophenolate Mofetil<br>• Pentostatin<br>• Prednisone<br>• Rituximab<br>• Sirolimus<br>• Tacrolimus<br>• TKI (i.e. imatinib)<br>• Other JAK inhibitor<br>• Other	• CS initiated or CS dose increased after ruxolitinib
Please specify the overall response to the most recent line of treatment patient has received according to NIH Consensus for measuring Therapeutic response[1]<br>Dropdown menu:<br>• CR: Complete response in all organs<br>• PR: Partial response in at least one organ, no progression in others<br>• MR: Improvement in at least one organ plus worsening in at least one organ<br>• PD: Progression in at least one organ	Please provide systemic therapy(ies) used at present to treat cGvHD (in addition to ruxolitinib). Multiselect list (multiple options can be selected):<br>• Methylprednisolone<br>• Prednisone<br>• Dexamethasone<br>• ATG<br>• Aldesleukin (IL-2)<br>• Azathioprine

• SD: no change	• Bortezomib<br>• Cyclosporine<br>• ECP<br>• Etanercept<br>• Tacrolimus<br>• Ibrutinib<br>• IL inhibitors<br>• Infliximab<br>• Methotrexate<br>• Mycophenolate Mofetil<br>• Pentostatin<br>• Rituximab<br>• Sirolimus<br>• TKI (i.e. imatinib)<br>• Other JAK inhibitor<br>• Other
What is the overall severity of cGvHD at present (NIH)[1]?<br>Drop-down menu:<br>• Mild: 1 or 2 organs involved with no more than score 1 plus lung score 0<br>• Moderate: 3 or more organs involved with no more than score 1 OR at least 1 organ (not lung) with a score of 2 OR lung score 1	

• Severe: At least 1 organ with a score of 3 OR lung score of 2 or 3<br>• None	

[1] Harris AC, et al. International, Multicenter Standardization of Acute Graft-versus-Host Disease Clinical Data Collection: A Report from the Mount Sinai Acute GVHD International Consortium. *Biol Blood Marrow Transplant*. 2016 Jan;22(1):4–10.

[2] Lee SJ, et al. Measuring therapeutic response in chronic graft-versus-host disease. National Institutes of Health consensus development project on criteria for clinical trials in chronic graft-versus-host disease: IV. The 2014 Response Criteria Working Group report. *Biol Blood Marrow Transplant*. 2015; 21:984–99.

[3]Jagasia MH, et al. National Institutes of Health Consensus Development Project on Criteria for Clinical Trials in Chronic Graft-versus-Host Disease: I. The 2014 Diagnosis and Staging Working Group report. *Biol Blood Marrow Transplant.* 2015 Mar;21(3):389-401.e1.

aGvHD, acute graft versus host disease; ATG, anti-thymocyte globulin; cGvHD, chronic graft versus host disease; CR, complete response; CS, corticosteroid(s); ECP, extracorporeal photopheresis; GI, gastrointestinal; IL, interleukin; JAK, Janus kinase; MR, mixed response; NIH, National Institutes of Health; PD, progressive disease; PR, partial response; SD, stable disease; TKI, tyrosine kinase inhibitor; TNF, tumor necrosis factor
